# Time-Shift Multiscale Fuzzy Entropy and Laplacian Support Vector Machine Based Rolling Bearing Fault Diagnosis

**DOI:** 10.3390/e20080602

**Published:** 2018-08-13

**Authors:** Xiaolong Zhu, Jinde Zheng, Haiyang Pan, Jiahan Bao, Yifang Zhang

**Affiliations:** 1 School of Mechanical Engineering, Anhui University of Technology, Maanshan 243032, China; 2 Engineering Research Center of Hydraulic Vibration and Control, Ministry of Education, Maanshan 243032, China

**Keywords:** multiscale entropy, fuzzy entropy, time-shift multiscale fuzzy entropy, rolling bearing, fault diagnosis, Laplacian support vector machine

## Abstract

Multiscale entropy (MSE), as a complexity measurement method of time series, has been widely used to extract the fault information hidden in machinery vibration signals. However, the insufficient coarse graining in MSE will result in fault pattern information missing and the sample entropy used in MSE at larger factors will fluctuate heavily. Combining fractal theory and fuzzy entropy, the time shift multiscale fuzzy entropy (TSMFE) is put forward and applied to the complexity analysis of time series for enhancing the performance of MSE. Then TSMFE is used to extract the nonlinear fault features from vibration signals of rolling bearing. By combining TSMFE with the Laplacian support vector machine (LapSVM), which only needs very few marked samples for classification training, a new intelligent fault diagnosis method for rolling bearing is proposed. Also the proposed method is applied to the experiment data analysis of rolling bearing by comparing with the existing methods and the analysis results show that the proposed fault diagnosis method can effectively identify different states of rolling bearing and get the highest recognition rate among the existing methods.

## 1. Introduction

Rolling bearing is a typical part for realizing the rotational motion and also prone to broken in the rotating machinery due to the complexity of working conditions, which will result in the machinery operation unstable or even unexpected accidents. Hence, it is of significance to timely recognize the faults for avoiding the loss of machinery life and factory finance. Generally, when the rolling bearing works with local faults, the non-linearity and non-stationarity are obviously expressed in vibration signals, which makes the traditional linear analysis methods loss their functions in analyzing these kinds of signals [1,2,3].

At present, many nonlinear dynamic analysis methods, such as approximate entropy (ApEn) [4,5], sample entropy (SampEn) [6,7], fuzzy entropy (FuzzyEn) [8,9] and multiscale entropy (MSE) [10,11] have been proposed and applied to the complexity measurement of the time series from different domains. Compared with traditional linear analysis methods, the nonlinear dynamic methods can effectively extract the nonlinear fault features from vibration signals of rolling bearings and promote the performance on fault identification and detection of rolling bearing [12]. For example, based on lifting wavelet packet transform and sample entropy as the method for extracting feature as well as binary tree system based classifier ensemble as the classification method, a new fault diagnosis method for bearing was proposed to automatically identify different fault types and severity levels in [13] and the experimental data analysis validated the more accuracy and stability performance of the proposed method on the fault recognition for rolling bearing. By combining empirical mode decomposition and sample entropy, a fault diagnosis method for rolling bearings was put forward by Zhong et al. in [14] and the results indicated that the feature information of intrinsic mode functions (IMFs) can be effectively extracted by calculating sample entropy and applied to fault diagnosis of rolling bearing. Chen et al. developed FuzzyEn as an enhanced algorithm of SampEn and ApEn and the analysis on electromyography (EMG) experimental data indicated that compared with SampEn and ApEn, FuzzyEn has more stronger relation consistence and little dependence on data length by using fuzzy function to measure the similarity of two vectors [15]. However, the entropy under unitary scale calculated from the original series is often insufficient for indicating the complexity of time series. To overcome this, MSE was put forward and developed in [16] by combing the coarse-grained method of time series. In [17], the MSE and adaptive neuro-fuzzy inference based fault diagnosis method for rolling bearing was proposed and the researches indicated that comparing the single scale-based entropy, MSE can be utilized to effectively extract the nonlinear, interaction and coupling characteristic contained in the mechanical signals and the proposed method could get a better fault recognition performance on bearing incipient fault diagnosis. Also, the anomaly detection method based on MSE and principal component analysis was proposed [18] and the simulation results suggested that MSE can be used to detect gear tooth pitting. However, the MSE method still has some intrinsic drawbacks. Firstly, the coarse-grained method in nature is a linear filter and the insufficient coarse-grained procedure will result in missing of pattern information. Secondly, with the increasing of time scale factors, the SampEns at larger factors will fluctuate heavily and even have no definitions.

By combing the FuzzyEn proposed by Chen et al. [19] and inspired by the idea of time shift way [20], a new complexity measuring method of time series called time shift multiscale fuzzy entropy (TSMFE) is proposed in this paper to overcome the above limitations of MSE. Then TSMFE is compared with MSE and time shift sample entropy (TSME) by simulation signal analysis and is applied to the complexity feature extraction of vibration signals from faulty rolling bearings. After obtaining the fault features by using TSMFE, it is necessary to select an intelligent classifier to automatically identify the fault types and degrees. Support vector machines (SVM) have been widely used in various pattern classification researches, such as face and figure recognition, fault classification and so on [21,22,23]. However, SVM requires that the class labels of all training samples should be known. In the real world, lots of fault samples are easily collected but hardly marked. Combining the manifold learning and SVM, a semi-supervised learning method called Laplacian support vector machine (LapSVM) was developed [24,25]. Compared with SVM, LapSVM requires less marked samples for learning [26]. A new fault diagnosis method for rolling bearings was proposed by combining TSMFE with LapSVM and then was compared with the existing methods by analyzing the experiment data of rolling bearing and the results verified its effectiveness.

The contents of this paper are constructed as follows. In Section 2, MSE and TSME algorithms are reviewed and then TSMFE is proposed for complexity measure of time series. The comparison analysis for 1/*f* noise and Gaussian white noise is given in Section 3. In Section 4, the new fault diagnosis method for rolling bearing is proposed by combining TSMFE with LapSVM and applied to the experimental data analysis of rolling bearing. Finally, the conclusions are given in Section 5.

## 2. Time Shift Multiscale Fuzzy Entropy and Related Theories

### 2.1. Multiscale Entropy Method

The MSE method can be calculated as follows [27,28,29].

For a determined number $\tau_{\max}$, the initial time series $X:x_{1},x_{2},\cdots ,x_{N}$ can be reconstructed as

(1) $$y_{j}(\tau )=\frac{1}{\tau }\sum_{i=(j-1)\tau +1}^{j\tau }x_{i},(1\leq j\leq N/\tau ,\tau =1,2,\cdots ,\tau_{\max}),$$

where τ is positive integer termed scale factor. The reconstructed time series is the original time series when τ=1 in particular and in case τ≥2, the reconstructed time series $y_{j}(\tau )$ is called coarse-grained time series with length no more than N/τ.

For each scale factor τ, the SampEn of $y_{j}(\tau )$ is respectively calculated and MSE is expressed as

(2) $$MSE(m,r,N,\tau )=\underset{\tau =1,2,\cdots ,\tau_{\max}}{SampEn}(m,r,N)=\left[-\ln\frac{B^{m+1}(r)}{B^{m}(r)}\right],$$

Actually, the coarse-grained process in MSE is regarded as a linear filter. The insufficient coarse-grained procedure will lead to the loss of pattern information hidden in time series. To surmount this problem, based on Higuchi’s fractal dimension (HFD) theory, time shift multiscale sample entropy was proposed by Tuan [20,30] by redefining the coarse-grained procedure and was applied to process physiological signals.

### 2.2. Time Shift Multiscale Sample Entropy

Based on HFD theory, time shift multiscale sample entropy [20] is described as follows.

(1) For the given time series $X=\left\{x_{1},x_{2},\cdots ,x_{N}\right\}$ with length *N*, the new time series can be obtained by

(3) $$Y_{k}^{\beta }=(x_{\beta },x_{\beta +k},x_{\beta +2k},\cdots ,x_{\beta +\Delta_{\left(\beta ,k\right)}k}),$$

where *k* and β(β=1,2,⋯,k) are positive integer, standing for the initial time point and interval time, and in particular, $\Delta_{\left(\beta ,k\right)}$ is a rounding integer for (N−β)/k, and represents the upper boundary.

(2) For each τ, the average of the SampEns of all time shift time series under 1≤β≤k is expressed as $TSME_{k}^{\beta }$ under single scale (namely k=τ), and for each *k* (1≤k≤τ) the $TSME_{k}$ with multiple scale can be described as

(4) $${\underset{1\leq k\leq \tau }{TSME}}_{k}=\frac{1}{k}\sum_{\beta =1}^{k}TSME_{k}^{\beta }.$$

TSME has overcame the drawbacks of MSE that the insufficient coarse graining will lead to the loss of patter information and however, some shortcomings remain to exist. In sample entropy used in MSE and TSME, the function for measuring similarity of two vectors cannot effectively recognize the two vectors with fuzzy boundaries in real world and the entropies will fluctuate heavily and even more have no definition with the increase of scale factor. To overcome this, TSMFE is proposed by combing TSME with FuzzyEn, by which the similarity of two vectors can be measured more robust than SampEn used in TSME and MSE.

### 2.3. Time Shift Multiscale Fuzzy Entropy

According to TSME and FuzzyEn [19], the detailed procedures of the proposed TSMFE method can be described as follows.

**Figure 1:**
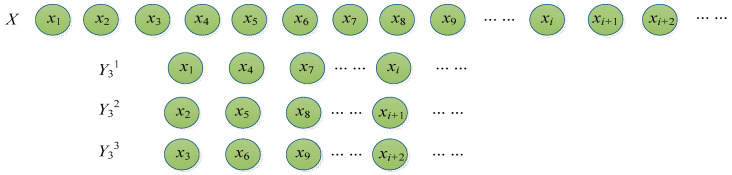
The procedure for reconstructing the time series.

(1) For the given time series $X=\left\{x_{1},x_{2},\cdots ,x_{N}\right\}$, it can be generated by using Equation (3). Particularly, when time interval k=3, the new time series is illustrated in Figure 1.

(2) For a determined time scale τ, the average of FuzzyEns of all time-shift time series under 1≤β≤k is expressed as $TSMFE_{k}^{\beta }$ (namely k=τ), and for each *k* (1≤k≤τ) the $TSMFE_{k}$ with multiple scale can be described as

(5) $${\underset{1\leq k\leq \tau }{TSMFE}}_{k}=\frac{1}{k}\sum_{\beta =1}^{k}TSMFE_{k}^{\beta }.$$

TSMFE is a nonlinear dynamic method for complexity measurement of time series. The comparison will be made in the following sections to verify the superiority of TSMFE.

## 3. Comparison of TSME and TSMFE

### 3.1. Parameter Selection

As shown in Equation (5), the calculation of TSMFE is relative with the embedding dimension *m*, similarity tolerance *r*, gradient parameter *n* and time series length *N*. Firstly, with the increase of *m*, the more feature information will be extracted from the reconstructed time series, however, a much longer time series is needed (generally $N=10^{m}\sim 30^{m}$). Hence, generally, we set *m* as 2. Secondly, the similarity tolerance *r* stands for the boundary width of fuzzy function and a too smaller *r* will result in too much useless information counted and the entropy will be sensitive to noise, while too larger *r* will lead to the loss of much statistical characteristics. Thus generally *r* ranges from 0.1SD to 0.25SD (SD is the standard deviation of the original time series) and 0.15SD is set in this paper. Thirdly, gradient parameter *n* controls the similarity of two vectors and when *n* tends to infinite, the exponential function becomes the unit step function which makes much statistical information missing. Hence, a smaller integer is suggested [19] and in this paper *n* is set as 2. Lastly, both the computation of SampEn and FuzzyEn over different scales are relative with data length and the literature [17] suggests that compared with SampEn, FuzzyEn need a shorter data length and thus in this paper *N* is set no less than 2000.

### 3.2. Simulation Analysis

**Figure 2:**
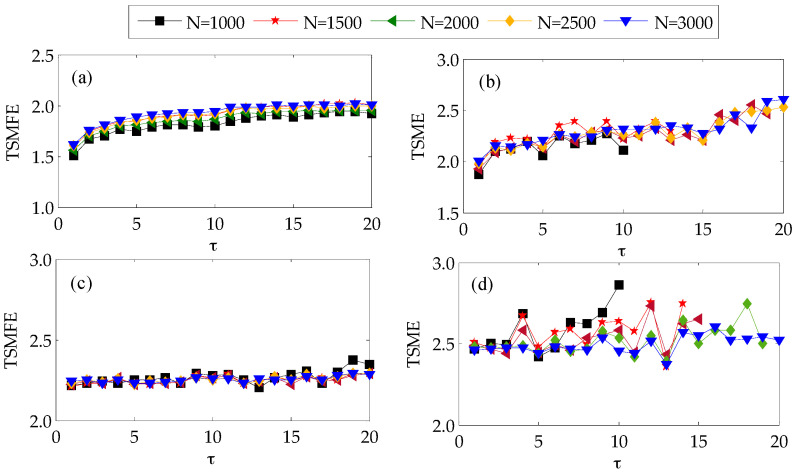
TSMFE and TSME of Gaussian white noises and 1/*f* noises under different lengths: (**a**) TSMFE of 1/*f* noise; (**b**) TSME of 1/*f* noise; (**c**) TSMFE of Gaussian white noise and (**d**) TSME of Gaussian white noise.

To illustrate the influence of data length on TSMFE, the Gaussian white noise and 1/*f* noise with data length 1000, 1500, 2000 and 25,000 are respectively used as examples with comparison with TSME, i.e., TSMEs and TSMFEs of the Gaussian white noises and 1/*f* noises with different length are calculated and the results are shown in Figure 2 where *m* = 2, *r* = 0.15SD, time internal *k* = 20 (that is τ=20). Firstly, as illustrated in Figure 2, both TSMEs and TSMFEs of 1/*f* noise gradually increase with the increase of scale factor, while TSMEs and TSMFEs of white noise almost remain to be a constant value which generally is larger than that of 1/*f* noise at most scales. This indicates that it is of importance to conduct multiscale analysis for time series. Secondly, for the time series under a larger time scale, i.e., with a shorter data length, TSME will have no definition. By contrast, TSMFEs of 1/*f* noise and white noises vary slightly with the increase of data length and the change of data length cannot lead to no definition of all time scales, which indicates that compared with TSME, data length has little influence on TSMFE. The above analysis indicates that both TSMFE and TSME can reflect the pattern information and complexity of the two kinds of random signals. By observing the TSME and TSMFE curves of Gaussian white noise and 1/*f* noise, we can find that TSMFE is more stable than TMSE especially in analyzing a shorter time series.

**Figure 3:**
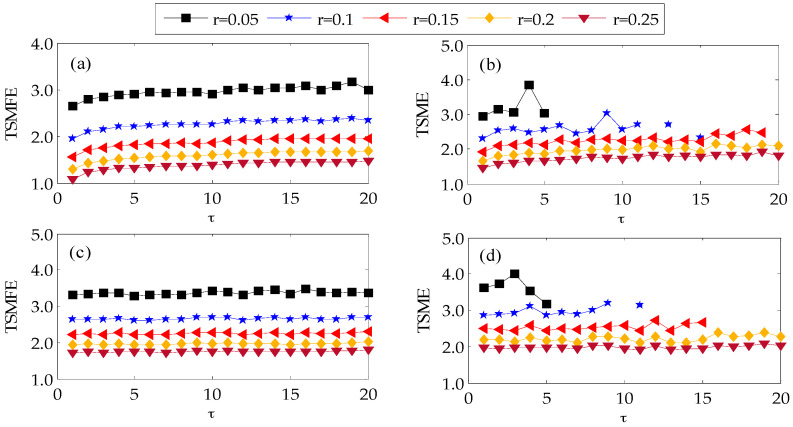
TSMFE and TSME of Gaussian white noise and 1/*f* noise under different similar tolerance: (**a**) TSMFE of 1/*f* noise; (**b**) TSME of 1/*f* noise; (**c**) TSMFE of Gaussian white noise and (**d**) TSME of Gaussian white noise.

To investigate the influence of similarity tolerance on TSMFE and TSME, the TSME and TSMFE of Gaussian white noises and 1/*f* noises with *N* = 2048 are calculated under different similarity tolerances *r* = 0.05SD, 0.1SD, 0.15SD, 0.2SD and 0.25SD (where *m* = 2, τ=20) and the results are shown in Figure 3. From the Figure 3, it can be found that for the same similarity tolerance *r*, with the increase of scale factor, the TSMFEs of 1/*f* noise and white noise vary slightly and even tend to a constant, while TSMEs of the two kinds of noises fluctuate obviously. Besides, with the increase of similarity tolerance *r*, the TSMFE and TSME values under the same time scales decrease gradually. However, for a smaller similarity tolerance, TSMEs have no definition for a larger scale factor while TSMFEs are always meaningful. Since a larger *r* will cause much statistic information missing while a smaller *r* will result in more unexpected statistical information, generally, we set *r* = 0.15SD. Therefore, the above analysis indicate that the TSMFE curves of two kinds of noises are more smooth and have smaller standard deviation than that of TSME at different similar tolerance.

## 4. TSMFE and LapSVM Based Fault Diagnosis Method for Rolling Bearing

### 4.1. LapSVM Algorithm

A better classifier is needed to fulfill an intelligent identification and classification. SVM, as a supervised learning method, has been used in many areas [31,32,33]. However, in SVM too many samples called training data should be labeled, which is difficult to obtain for the real world data. To overcome this drawback of SVM, Combining the manifold regularization, Laplacian support vector machine (LapSVM), a semi-supervised learning, has been proposed and wildly applied on feature recognition [34,35,36]. In LapSVM, the recognition performance can be promoted by manifold regularization and thus much unmarked data are used to estimate the internal data manifold structure that will be applied to design the classifier.

When a set of marked samples $\left(x_{i},y_{i}^{E}\right)$ (i=1,2,⋯,l) and kernel function *K* are determined, and the hinge loss function is selected as loss function, the manifold regularization framework can be expressed as

(6) $$f^{*}=\arg\min\frac{1}{l}\sum_{i-1}^{l}\left(1-y_{i}^{E}f(x_{i})\right)+\gamma_{A}{\left\|f\right\|}_{k}^{2}+\frac{\gamma_{I}}{{\left(u+l\right)}^{2}}F^{T}LF,$$

where $F={\left[f(x_{1}),f(x_{2}),\cdots ,f(x_{l+u})\right]}^{T}$, *l* and *u* respectively stand for the number of given marked samples $\left(x_{i},y_{i}^{E}\right)$ and unmarked samples $x_{j}$, (j=l+1,l+2,⋯,l+u), manifold regularization item is denoted as ${\left\|f\right\|}_{I}^{2}$, both $\gamma_{I}$ and $\gamma_{A}$ are manifold regularization parameter, *L* represents Laplacian matrix, hinge loss function $V(x_{i},y_{i}^{E},f)={\left(1-y_{i}^{E}f(x_{i})\right)}_{+}=\max(0,1-y_{i}^{E}f(x_{i}))$ is used to measure the deviation of the desired outputs $y_{i}^{E}\in \left\{-1,1\right\}$ and real outputs $f(x_{i})$ of training samples $x_{i}$. Combining express theorem, the Equation (6) is solved as

(7) $$f^{*}=\sum_{i=1}^{l+u}a_{i}^{*}K(x,x_{i}),$$

when $\alpha *={\left[\alpha_{1},\alpha_{2},\cdots ,\alpha_{l+u}\right]}^{T}$,

(8) $$\alpha^{*}={\left[2\gamma_{A}I+2\frac{\gamma_{1}}{{\left(u+l\right)}^{2}}LK\right]}^{-1}J^{T}Y\beta^{*},$$

in which $I_{{}_{\left(l+u)(l+u\right)}}$ and $L_{\left(l+u\right)\left(l+u\right)}$ are respectively unit matrix and Laplacian matrix, nuclear matrix is denoted as $K_{\left(l+u)(l+u\right)}$, $Y=diag(y_{1}^{E},y_{2}^{E},\cdots ,y_{l}^{E})$ is used to balance the complexity of the experience loss and function, by quadratic planning $\beta^{*}$ is expressed as

(9) $$\beta^{*}=\underset{\beta \in R^{l}}{\max}\sum_{i=1}^{l}\beta_{i}-\frac{1}{2}\beta^{T}Q\beta ,$$

when $\sum_{i=1}^{l}\beta_{i}y_{i}^{E}=0(0\leq \beta_{i}\leq 1/l,i=1,\cdots ,l)$,

(10) $$Q=YJK{\left(2\gamma_{A}I+2\frac{\gamma_{1}}{{\left(I+u\right)}^{2}}LK\right)}^{-1}J^{T}Y.$$

Based on the above analysis, the unmarked samples used in LapSVM can be employed to estimate the internal manifold structure used to the aided learning of marked samples, which obviously promotes the recognition performance, while in SVM all the samples should be labeled. Considering the difficulty of sample to be labeled in the real world, LapSVM is applied in this paper.

### 4.2. The Proposed Fault Diagnosis Method

The above analysis indicate that TSMFE, as a new complexity and irregularity measuring method of time series, can get much better performance than TSME. Meanwhile, the data length and similar tolerance also have less influence on TSMFE than TSME and the entropies, obtained by TSMFE, are also more stable. Hence, TSMFE is employed to the fault feature extraction and diagnosis for rolling bearing.

The proposed fault diagnosis method for rolling bearing is described as follows:

(1) For given *p* kinds of states of rolling bearing, each state has *m_p_* samples and thus the number of whole samples is $M=(\sum_{i=1}^{p}m_{i})$;
(2) TSMFE of all the *M* samples are calculated and the feature sets $\left(T^{p},p\right)$, $T^{p}\in R^{m_{p}\times \tau_{\max}}$ are obtained, where $T^{p}$ represents the *p*-th feature sets;
(3) The *m_p_* samples of the *p*-th state are randomly divided into h as marked sample sets, i.e., $T_{1}^{p}\in R^{h\times \tau_{\max}}$ and (*m_p_* − *h*) as unmarked sample sets, i.e., $T_{2}^{p}\in R^{(m_{p}-h)\times \tau_{\max}}$;
(4) The sensitive fault features sets of training samples: both $T_{1}^{p}\in R^{h\times \tau_{\max}}$ and $T_{2}^{p}\in R^{(m_{p}-h)\times \tau_{\max}}$ are input to the LapSVM classifier for training, learning and testing.

**Figure 4:**
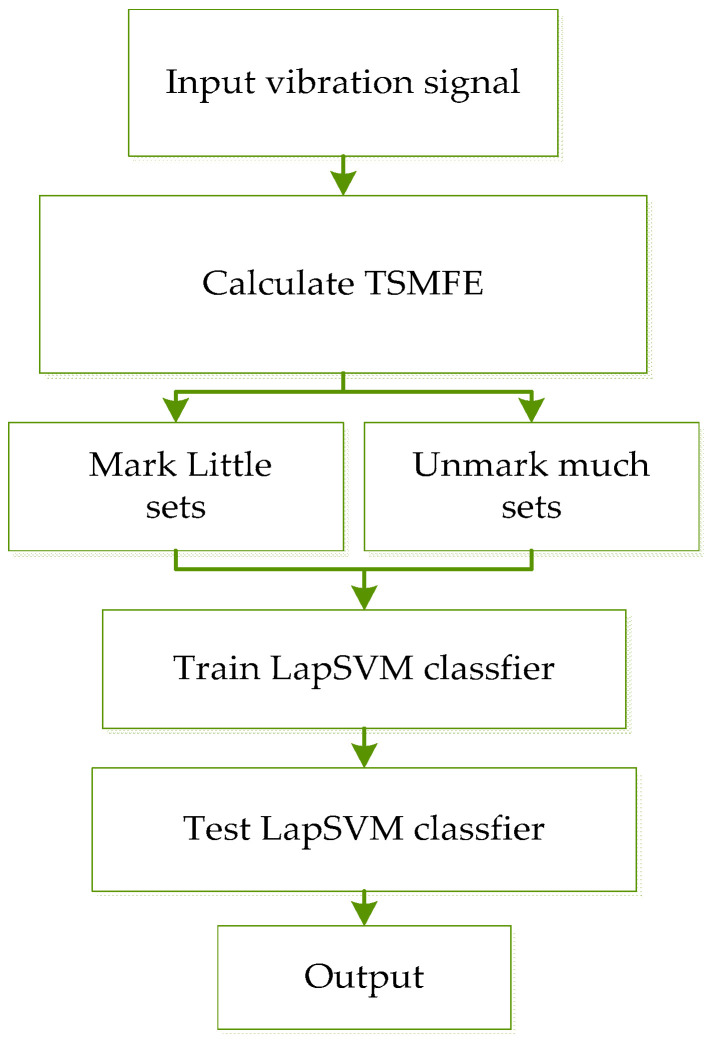
The proposed fault diagnosis procedure for rolling bearing.

The procedure of the proposed method can be described as follows (Figure 4).

### 4.3. Experimental Data Analysis

**Figure 5:**
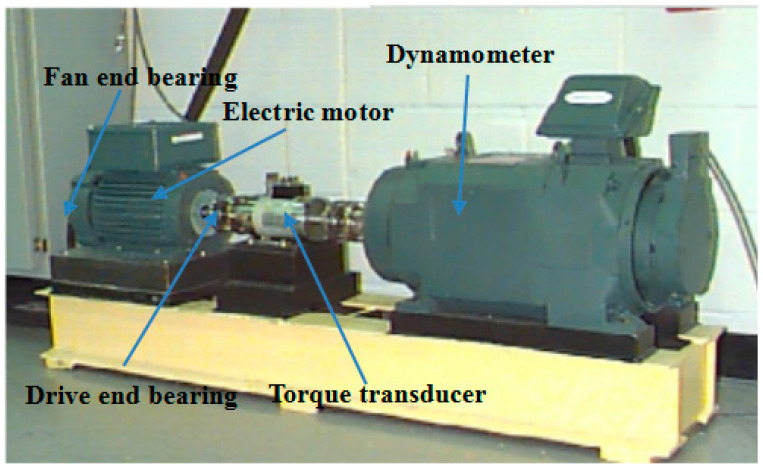
The test stand of CWRU.

**Figure 6:**
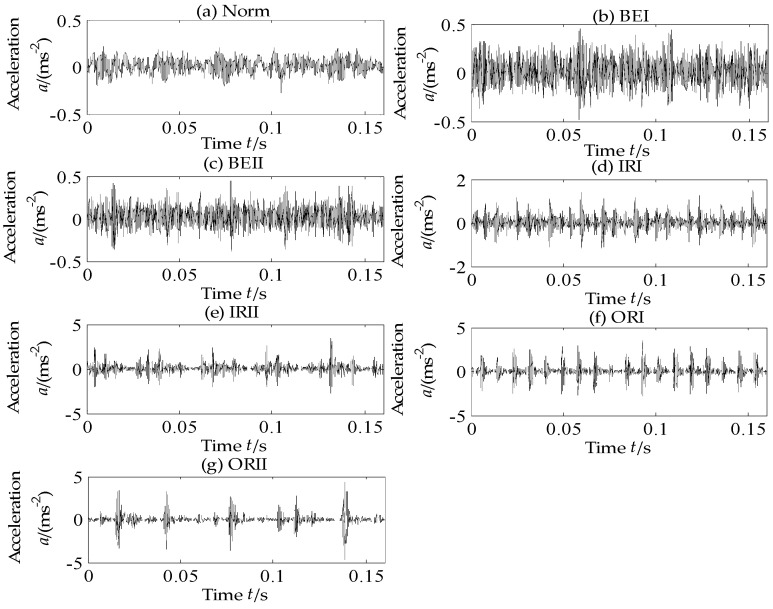
Time-domain vibration signals of seven states of rolling bearing.

In this subsection, the experimental data supported by Case Western Reserve University [37] are employed to verify the effectiveness of the proposed method. As shown in Figure 5, the experiment stand is composed of fan end bearing, drive end bearing, torque transducer and dynamometer. The type of tested rolling bearing is 6205-2RS JEM SKF (SKF, Göteborg, Sweden), in which single point faults have been machined by electro-discharge. Particularly, the fault diameters are respectively 0.1778 mm, 0.3556 mm and 0.5334 mm with depth 0.2794 mm. There are four states for rolling bearing, i.e., Normal (Norm), Ball element default (BE), Inner race default (IR) and Outer race default (OR). The vibration acceleration signals of rolling bearing under four states have been respectively collected when the motor speeds are 1797 r/min, 1772 r/min, 1750 r/min, and 1730 r/min. The sensors are fixed at fan end with sampling frequency 12 kHz and drive end with sampling frequency 12 and 48 kHz. In this paper, the motor speed is 1797 r/min without load, the acceleration signals of rolling bearings on normal state and three fault states under two fault diameters: 0.1778 mm and 0.5334 mm are collected from drive end with sampling frequency 12 kHz. They are successively denoted as: (a)normal state (Norm), (b) ball element fault with fault diameters: 0.1778 mm(BEI), (c) ball element fault with fault diameters: 0.5334 mm (BEII), (d) inner ring fault with fault diameters: 0.1778 mm (IRI), (e) inner ring fault with fault diameters: 0.5334 mm (IRII), (e) outer ring fault with fault diameters: 0.1778 mm (ORI) and (f) outer ring fault with fault diameters: 0.5334 mm (ORII). All the time-domain vibration signals of the seven states of rolling bearings are shown in Figure 6 and it is of difficulty to entirely distinguish the states of rolling bearing from the time-domain signals.

**Figure 7:**
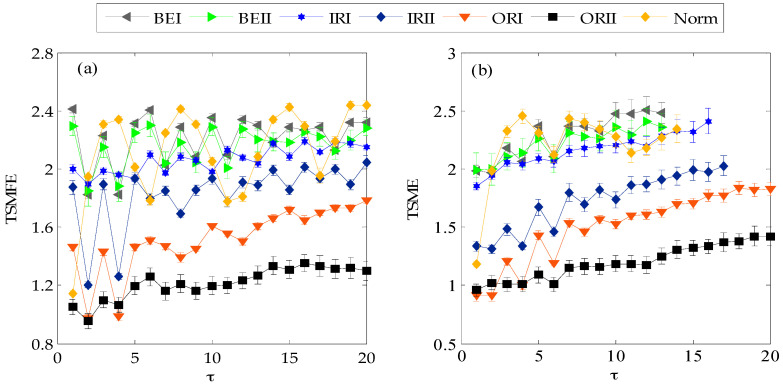
Mean and standard deviation of TSMFE and TSME: (**a**) mean and square deviation of TSMFE; (**b**) mean and square deviation of TSME.

Next, 50 samples with length 2048 of each state of rolling bearing were used to test the performance of TSMFE in vibration signal analysis of rolling bearing. TSMFEs and TSMEs of all the selected samples were respectively calculated and exhibited in Figure 7. It can be seen that with the increase of scale factor, TSMEs of vibration signals of rolling bearing under several categories have no definition at the larger scale factor and this indicates that TSMFE is more stable and effective to represent the pattern features than TSME. Besides, at the same time scale, the TSMFEs of vibration signals of rolling bearing fluctuate slighter than that of TSME and it is beneficial to promote the identification performance. Finally, as shown in Figure 7, TSMFEs and TSMEs of vibration signals of rolling bearing with ball element fault are generally larger than that of rolling bearing with inner ring fault, which are larger than that of rolling bearing with outer ring faults at the most of scale factors. The above analysis indicates that compared with TSME of vibration signals of rolling bearing, TSMFE can represent the fault feature more effectively and get much better performance on the fault diagnosis of rolling bearing.

**Figure 8:**
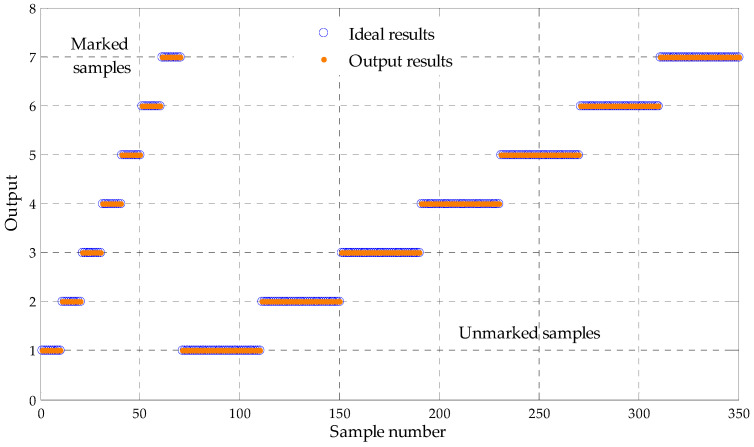
Output results of the LapSVM based multi-classifier of test samples.

Next, 50 samples of each states are randomly divided into 10 marked training sample feature sets and 40 unmarked testing ones. The sensitive fault features subset of samples are correspondingly input to the LapSVM based multi-fault classifier, where the radial basis function is selected as the kernel function of LapSVM, and the parameter of the kernel function is set to 0.35. The binary tree theory is adopted to construct the multi-fault classifier. The detailed results are shown in Table 1 and Figure 8. As shown in Table 1 and Figure 8, in LapSVM1, LapSVM2, LapSVM3, LapSVM4, LapSVM5 and LapSVM6, all samples are accurately identified and the total identification rate is 100%, which validates that the proposed method for fault diagnosis of rolling bearing has a well recognition performance.

**Figure 9:**
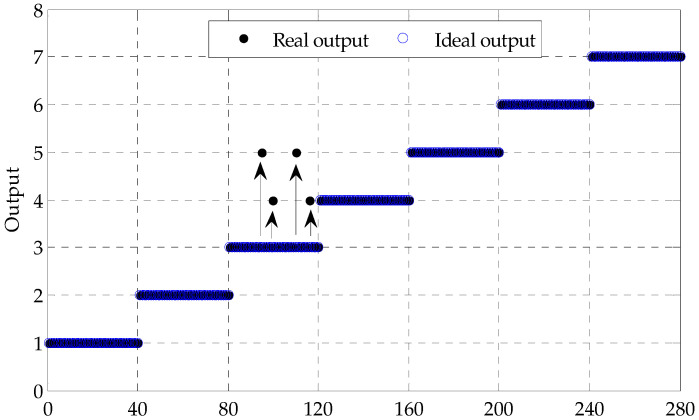
Output results of the SVM based multi-classifier of test samples.

The above obtained and marked samples also are input to the SVM based multiple fault classifier with the same parameters set in LapSVM. The outputs are shown in Table 2 and Figure 9. As shown in Table 2 and Figure 9, the recognition rate is 98.57% in which four samples with BEII are wrongly divided into that with IRI and IRII respectively and equally in SVM3. Eventually, comparing Table 1 and Table 2, the fault type of rolling bearing can be effectively classified by LapSVM when much unmarked samples are adopted, which indicates that the proposed method has a better recognition performance on fault diagnosis of rolling bearing.

**Figure 10:**
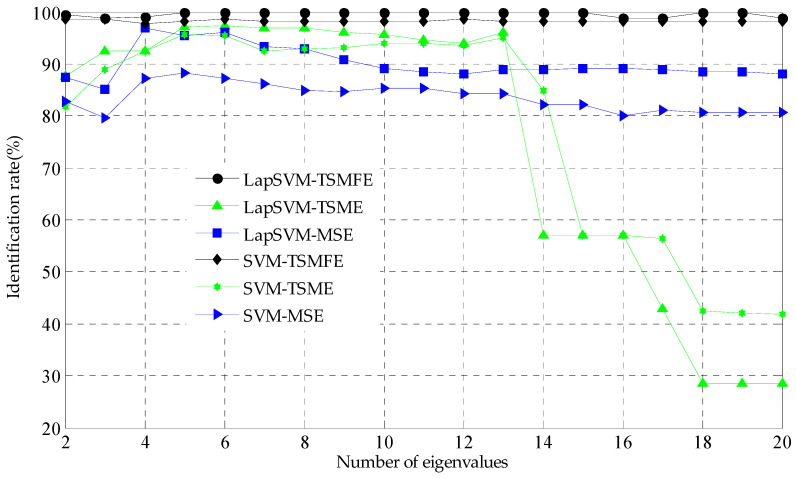
Identification rate comparison of the TSMFE-, TSME- and MSE-based methods.

To illustrate the superiority of TSMFE, for comparison, TSME and MSE methods are also used to extract the feature information by analyzing the above experimental data of rolling bearing. TSMFEs, TSMEs and MSEs of all selected vibration signals of rolling bearing are calculated and input to the LapSVM multi-classifier for multi-fault recognition of rolling bearing. The recognition results are shown in Figure 10, where the number of marked samples is ten and the number of input features are varying from 2 to 20. Firstly, when the number of marked samples is determined, with the increase of the number of selected features, the recognition rate of the MSE, TSME and TSMFE based fault diagnosis methods all increase and then decrease to some degree, which means that not all of entropies in MSE, TSME and TSMFE are suitable to reflect the fault feature of rolling bearing and some of them are redundant information. Secondly, compared with the existing methods, it is obvious that the rate recognition of the proposed method fluctuates slightly and tend to be 100%, which are always larger than that of the TSME and LapSVM based fault methods for different numbers of features, as well as the MSE and LapSVM based fault diagnosis method when the number of input features is smaller than 14. Particularly, when the number of input features is larger than 14, the recognition performance of the TSME and LapSVM method is decreasing heavily and this is because that the MSE has no definition at the larger scale factor and the LapSVM cannot classify the fault feature effectively. Although TSME overcomes the obstacles of MSE that with the increase of time scales, the entropies in TSME tend to no definition to some degree, this drawback still exist. Finally, the above analysis has validated the superiority of TSMFE for extracting fault feature of rolling bearing. Compared with TSME and MSE, the number marked samples have little influence on the performance of TSMFE, which expresses much more stability. When the numbers are suitable, the identification rate of the proposed method for extracting fault features by TSMFE tends to 100% in particular.

**Figure 11:**
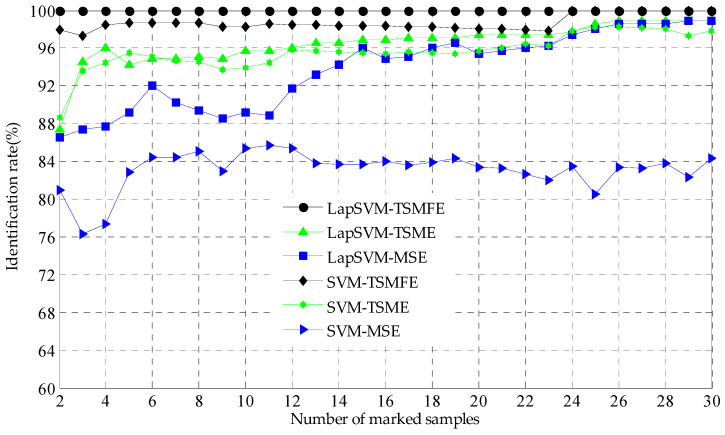
Identification rate comparison of the mentioned methods: TSMFE (or TSME and MSE) and LapSVM (or SVM).

Next, to illustrate the superiority of LapSVM, the above calculated entropies of TSMFE, TSME and MSE are input to SVM based multi-classifier for identification and the recognition results are shown in Figure 11, where the number of input features are set as 10 and that of the marked samples is varying from 2 to 30. With the increase of the number of marked samples, the recognition rates of the TSMFE and SVM based fault diagnosis method increase and tend to be a constant 100% and that of the TSME and SVM based fault diagnosis method increase with little fluctuation, while the recognition rates of the MSE and SVM based fault diagnosis method fluctuate severely tending to a constant 84%. In particular, the recognition rate of the TSMFE and SVM based fault diagnosis method is much larger than those of the TSME (or MSE) and SVM based methods. Secondly, with the increase of the number of marked samples, the recognition rates of the TSMFE and LapSVM based fault diagnosis method for rolling bearing remains 100%, which is larger than that of the TSME (or MSE) and SVM based methods. And thus the above analysis validates the superiority of TSMFE. Thirdly, with the increase of the number of marked samples, the recognition performance of LapSVM is always better than that of SVM for the same input features. Finally, by observing Figure 10 and Figure 11 we can conclude that the proposed TSMFE and LapSVM based fault diagnosis method for rolling bearing has much better fault recognition performance for rolling bearing than the contrasting methods.

Next, to illustrate the superiority of the proposed method, we also investigate the influence of the number of marked samples and input features on the fault recognition rates of the proposed method, as well as the existing methods. The recognition results for different methods are shown in Table 3 and Table 4 in detail, where the data in the first line stands for the number of marked samples and the first column represents the number of input features. Table 3 provides the recognition results of the LapSVM based fault diagnosis methods. When the number of marked samples is determined, with the increase of the number of extracted features, the recognition rates of the different methods i.e., MSE, TSME and TSMFE fluctuate obviously which indicates that the number of extracted features have some influence on the recognition performance. When the number of extracted eigenvalues is determined, with the increase of the number of marked samples, the recognition rate of the proposed method almost tends to be a constant that are larger than that of the MSE and TSME based fault diagnosis method with more obvious fluctuation, which means that the number of marked samples has little influence on the identification results of the proposed methods comparing the existing methods. The recognition results of the SVM based methods are shown in Table 4. Comparing with the MSE, TSME and SVM methods, the TSMFE and SVM based fault diagnosis method for fault diagnosis of rolling bearing has a higher recognition rater with little fluctuation than the existing methods which also validates the superiority of TSMFE to MSE and TSME. The comparing of Table 3 and Table 4 indicates the LapSVM based methods have higher identification performance than the SVM based methods. From the two tables, we can find that for the proposed method generally, we suggest the numbers of marked samples and input features should be ranging from 4 to 20 and from 8 to 12, respectively to obtain a better recognition performance. These two tables further indicate the superiority of the proposed methods.

## 5. Conclusions

In this paper, an improved algorithm of multiscale sample entropy (MSE) called TSMFE is proposed to measure the complexity of time series. The influence of similar tolerance and data length on TSMFE are investigated by analyzing Gaussian white noise and 1/*f* noise signals. The results indicate that compared with TSME, TSMFE obviously expresses more anti-noise property, more stable entropies even in shorter time series and slight fluctuation with the increase of time scale factors. Combining TSMFE and LapSVM, a new fault diagnosis method for rolling bearing is put forward and applied to analyze experimental data of rolling bearing. The comparison analysis of the proposed method with the existing methods, i.e., TSMFE with MSE and TSME, LapSVM with SVM are made and the analysis results validate that the proposed method has much better performance on fault recognition rate than the used comparison method. At present, the entropy theories have not been wildly used in mechanical signal processing for digging the nonlinear fault features hidden in the vibration signals. In this paper, we tried to apply the proposed TSMFE method to multi-fault diagnosis for rolling bearing and the simulation and experimental data analysis were also conducted. The following work will be emphasized on optimized feature selection and on-line multi-fault diagnosis.

## Figures and Tables

**Table 1:** Output results of LapSVM classifier.

Sample Sets	Faults	LapSVM1	LapSVM2	LapSVM3	LapSVM4	LapSVM5	LapSVM6
T1~T50	Norm	+1(50)					
T51~T100	BEI	−1(50)	+1(50)				
T101~T150	BEII	−1(50)	−1(50)	+1(50)			
T151~T200	IRI	−1(50)	−1(50)	−1(50)	+1(50)		
T201~T250	IRII	−1(50)	−1(50)	−1(50)	−1(50)	+1(50)	
T251~T300	ORI	−1(50)	−1(50)	−1(50)	−1(50)	−1(50)	+1(50)
T301~T350	ORII	−1(50)	−1(50)	−1(50)	−1(50)	−1(50)	−1(50)

**Table 2:** Output results of SVM classifier.

Sample Sets	Faults	SVM1	SVM2	SVM3	SVM4	SVM5	SVM6
T1~T50	Norm	+1(40)					
T51~T100	BEI	−1(40)	+1(40)				
T101~T150	BEII	−1(40)	−1(40)	+1(36)			
T151~T200	IRI	−1(40)	−1(40)	−1(42)	+1(40)		
T201~T250	IRII	−1(40)	−1(40)	−1(42)	−1(40)	+1(40)	
T251~T300	ORI	−1(40)	−1(40)	−1(40)	−1(40)	−1(40)	+1(40)
T301~T350	ORII	−1(40)	−1(40)	−1(40)	−1(40)	−1(40)	−1(40)

**Table 3:** Identification results with LapSVM (%).

	Methods	4	6	8	10	12	14	16	18	20	22
**4**	MSE	86.85	96.57	96.85	96.85	97.42	97.42	97.14	96.85	97.42	97.42
	TSME	86	91.43	92	92.57	93.43	96	95.71	96.29	96.29	96.87
	TSMFE	99.14	99.14	99.14	99.14	99.14	99.14	99.14	99.14	99.14	99.14
**8**	MSE	88.28	92.85	92.85	92.85	93.71	94.57	95.42	95.42	94.85	94.85
	TSME	96.29	96.57	96.29	96.86	97.14	97.43	97.43	97.71	97.71	97.71
	TSMFE	100	100	100	100	100	100	100	100	100	100
**12**	MSE	87.14	89.14	87.42	88	89.14	92.85	94.57	95.42	95.71	95.71
	TSME	92.85	93.42	93.42	94	94.28	94.57	95.14	95.71	96.57	96.28
	TSMFE	100	100	100	100	100	100	100	100	100	100
**16**	MSE	87.71	89.71	88.57	89.14	89.14	90.57	92.85	92.85	94	95.42
	TSME	57.14	57.14	57.14	57.14	57.14	57.14	57.14	57.14	57.14	57.14
	TSMFE	98.86	98.86	98.86	98.86	98.86	98.86	98.86	98.86	98.86	98.86
**20**	MSE	87.14	90	88.28	88	88.85	89.42	91.14	91.71	95.14	95.42
	TSME	28.57	28.57	28.57	28.57	28.57	28.57	28.57	28.57	28.57	28.57
	TSMFE	98.86	98.86	98.86	98.86	98.86	98.86	98.86	98.86	98.86	98.86

**Table 4:** Identification results with SVM (%).

	Methods	4	6	8	10	12	14	16	18	20	22
**4**	MSE	78.26	87.01	87.07	87.14	86.84	85.31	84.87	84.82	85.23	87.24
	TSME	86.95	93.18	93.87	92.50	90.97	92.85	91.59	92.41	93.80	93.36
	TSMFE	97.51	97.72	97.61	97.85	98.12	98.41	98.31	98.21	98.09	97.95
**8**	MSE	79.81	84.41	83.33	85.00	84.21	84.52	84.45	83.92	85.23	85.20
	TSME	95.03	95.45	94.21	92.85	95.48	96.03	95.79	96.42	96.66	96.42
	TSMFE	98.44	98.70	98.29	98.21	98.49	98.41	98.31	98.21	98.02	97.88
**12**	MSE	77.32	84.74	85.37	84.28	85.71	83.73	84.03	83.48	83.80	84.18
	TSME	93.16	94.15	94.55	93.57	94.73	95.23	94.95	95.53	96.19	95.91
	TSMFE	98.13	98.70	98.63	98.57	98.49	98.41	98.31	98.21	98.09	97.95
**16**	MSE	77.32	83.11	81.63	80.00	81.95	83.73	84.45	83.48	83.33	82.65
	TSME	79.81	57.14	57.14	57.14	57.14	57.14	68.48	57.14	57.14	57.14
	TSMFE	97.82	98.37	98.29	98.21	98.49	98.41	98.31	98.21	98.09	97.95
**20**	MSE	77.01	78.57	79.59	80.71	81.57	79.36	81.09	80.35	82.85	81.12
	TSME	47.20	41.88	41.83	41.78	47.74	41.66	41.59	28.57	28.57	36.22
	TSMFE	97.82	98.37	98.29	98.21	98.49	98.01	98.31	98.21	98.09	97.95

## Abbreviations

**Nomenclature**			
AppEn	Approximate entropy	τ	Time scale
SampEn	Sample entropy	*X*	Initial time series
MSE	Multiscale sample entropy	*N*	Length of data X
SVM	Support vector machine	m	Embedding dimension
LapSVM	Laplace support vector machine	*r*	Similar tolerances
FuzzyEn	Fuzzy entropy	*k*	Initial time point
TSMFE	Time shift multiscale fuzzy entropy	β	Interval time
TSME	Time shift multiscale entropy	$\Delta_{\left(\beta ,k\right)}$	Upper rounding boundary
Norm	Normal rolling bearing	*l*	Number of given marked samples
ORI	Outer race fault under fault diameters 0.1778 mm	*u*	Number of given unmarked samples
BEI	Ball element under fault diameters 0.1778 mm	${\left\|f\right\|}_{I}^{2}$	Manifold regularization item
IRI	Inner race fault under fault diameters 0.1778 mm	$f^{*}$	Manifold regularization framework
ORII	Outer race fault under fault diameters 0.5334 mm	$\beta^{*}$	Quadratic planning
BEII	Ball element under fault diameters 0.5334 mm	*p*	Number of rolling bearing states
IRII	Inner race fault under fault diameters 0.5334 mm	*M*	Total number of samples
SD	Standard deviation	$T^{p}$	*p*-th feature sets
*L*	Laplacian	$T_{1}^{p}$	Marked sample sets
*V*	Hinge loss function	$T_{2}^{p}$	Unmarked sample sets
